# Supplementary figures and images for: Understanding Epileptiform After-Discharges as Rhythmic Oscillatory Transients
Source: Front Comput Neurosci. 2017 Apr 18;11:25. doi: 10.3389/fncom.2017.00025 (PMC5394159; doi:10.3389/fncom.2017.00025)

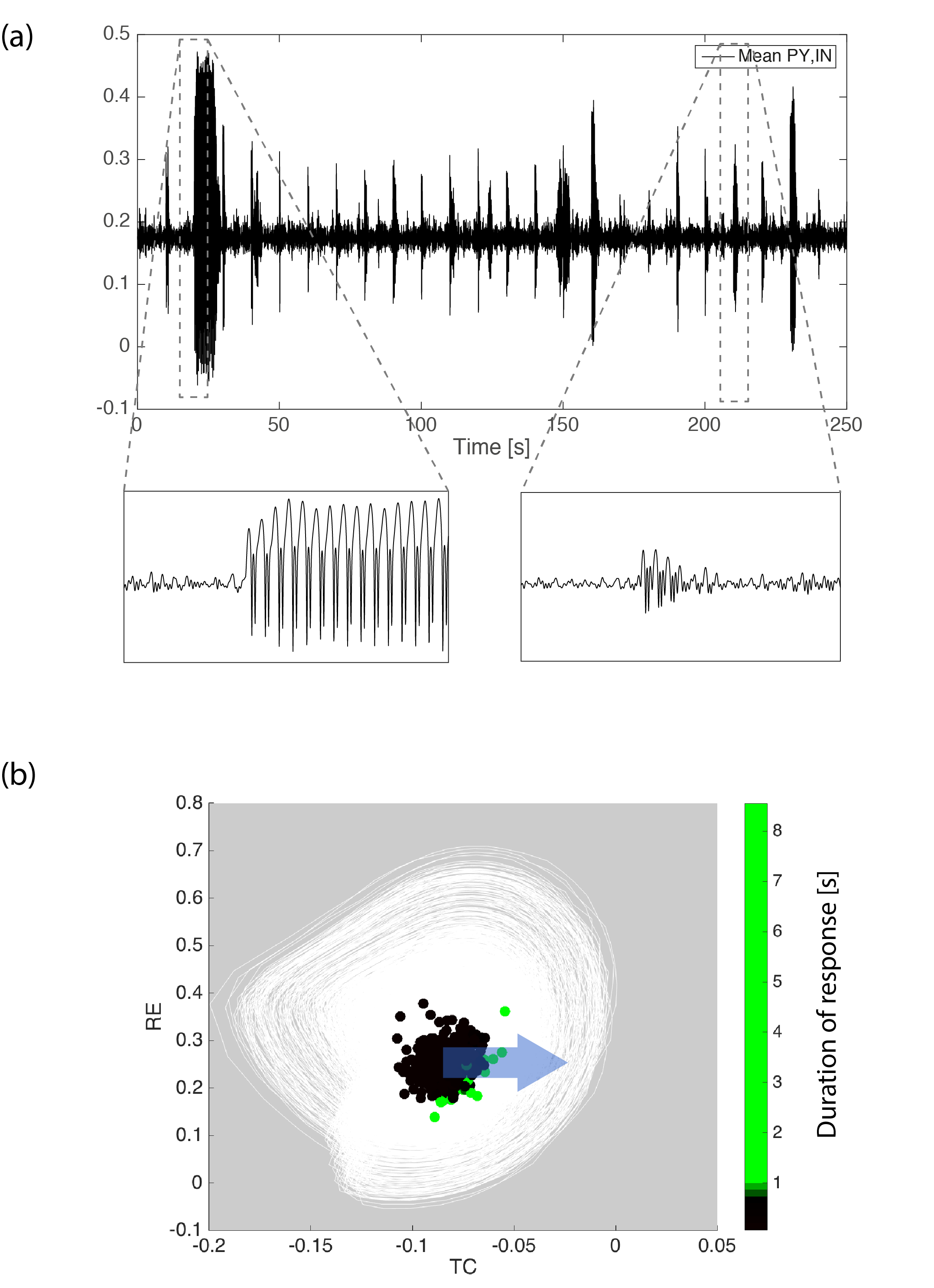

Supplement: Figure S1 — Afterdischarge duration depends on where in state space the system is when stimulated. (A) Simulation of repeated stimuli every 10 s to provoke afterdischarges in the 4V model with noise input. All parameters are unchanged between stimuli. (B) When plotting the time series from (A) in state space (white lines), we see the background state as a dense concentration of trajectories, and the stimulations occasionally cause bigger deviations beyond the background state. Overlayed, we show the state space position where stimuli were applied as dots. The color of the dots indicate the duration of the ensuing response. Larger deviations tend to cluster on the right hand side of the background state. The direction of simulated stimulation is shown with the blue arrow. [file Image1.PNG]
