# Supplementary material for: Understanding Epileptiform After-Discharges as Rhythmic Oscillatory Transients
Source: Front Comput Neurosci. 2017 Apr 18;11:25. doi: 10.3389/fncom.2017.00025 (PMC5394159; doi:10.3389/fncom.2017.00025)
Supplement: Table S1 — Parameter values used to produce the figures for the reduced 3D system in this manuscript. [file Table1.PDF]

Table S1: Parameter values used to produce the figures for the reduced 3D system in this manuscript.

Parameter	Interpretation	Fig. 3	Fig. 4a	Fig. 4b,c	Fig. 5a,b,c	Fig. 5d,e,f
C_a	$Z \rightarrow Y$ connectivity strength	2	2	2	2	2
C_b	$X \rightarrow Y$ connectivity strength	5	5	5	5	5
C_c	$Y \rightarrow Z$ connectivity strength	30	30	30	30	varies
C_d	$Y \rightarrow X$ connectivity strength	1	1	1	1	1
τ_{fast}	X timescale	23	23	23	23	23
τ_{slow}	Y, Z timescale	2.3	2.3	2.3	varies	2.3
h_X	Input X	-2	varies	-3.5	varies	varies
h_Y	Input Y	-3	-3	-3	-3	-3
h_Z	Input Z	3	3	3	3	3
ϵ	Sigmoid steepness	250000	250000	250000	250000	250000

Table S2: Parameter values used to produce the figures for the full thalamo-cortical system in this manuscript.

Parameter	Interpretation	Fig. 6	Fig. 7a,c	Fig. 7b,d	Fig. 7e,f	Fig. 8a,c	Fig. 8b,d
C_1	$PY \rightarrow PY$ connectivity strength	1.8	1.8	1.8	1.8	1.8	1.8
C_2	$PY \rightarrow IN$ connectivity strength	4	4	4	4	4	4
C_3	$IN \rightarrow PY$ connectivity strength	1.5	1.5	1.5	1.5	1.5	1.5
C_4	$RE \rightarrow RE$ connectivity strength	0.2	0.2	0.2	0.2	0.2	0.2
C_5	$TC \rightarrow RE$ connectivity strength	10.5	10.5	10.5	10.5	10.5	10.5
C_6	$RE \rightarrow TC$ connectivity strength	0.6	0.6	0.6	0.6	0.6	0.6
C_7	$PY \rightarrow TC$ connectivity strength	3	3	3	3	3	3
C_8	$PY \rightarrow RE$ connectivity strength	3	3	3	3	3	3
C_9	$TC \rightarrow PY$ connectivity strength	1	1	1	1	1	1
τ_1	PY timescale	26	26	26	26	26	26
τ_2	IN timescale	32.5	32.5	32.5	32.5	32.5	32.5
τ_3	TC timescale	2.6	2.6	2.6	2.6	2.6	2.6
τ_4	RE timescale	2.6	2.6	2.6	2.6	2.6	0.26
h_{py}	Input PY	-0.35	-0.35	-0.35	-0.35	-0.35	-0.35
h_{in}	Input IN	-3.4	-3.4	-3.4	-3.4	-3.4	-3.4
h_{tc}	Input TC	-2.0	varies	-2.1	-2.1	-2.1	-2.1
h_{re}	Input RE	-5	-4.85	varies	-4.85	-4.85	-5(b) -4.85(d)
ϵ	Sigmoid steepness	250000	250000	250000	250000	250000	250000
a	Linear intersection steepness	2.8	2.8	2.8	2.8	2.8	2.8
b	Linear intersection offset	0.5	0.5	0.5	0.5	0.5	0.5
